# Revealing the Pathogenesis of Salt-Sensitive Hypertension in Dahl Salt-Sensitive Rats through Integrated Multi-Omics Analysis

**DOI:** 10.3390/metabo12111076

**Published:** 2022-11-07

**Authors:** Ya-nan Ou-Yang, Meng-di Yuan, Zheng-mao Yang, Zhuo Min, Yue-xin Jin, Zhong-min Tian

**Affiliations:** 1 The Key Laboratory of Biomedical Information Engineering of Ministry of Education, School of Life Science and Technology, Xi’an Jiaotong University, Xi’an 710049, China; 2 Puripharm Co., Ltd., Huzhou 313000, China; 3 Department of Brewing Engineering, Moutai University, Renhuai 564500, China

**Keywords:** metabolic reprograming, gut-liver axis, gut microbiota analysis, salt-sensitive hypertension

## Abstract

Salt-induced renal metabolism dysfunction is an important mechanism of salt-sensitive hypertension. Given that the gut-liver axis is the first hit of a high-salt diet (HSD), we aimed to identify the extra-renal mechanism from hepatic metabolism and gut microbiota, and attempted to relieve the salt-induced metabolic dysfunctions by curcumin. Untargeted metabolomics analysis was performed to identify the changes in hepatic metabolic pathways, and integrated analysis was employed to reveal the relationship between hepatic metabolic dysfunction and gut microbial composition. HSD induced significant increase in fumaric acid, l-lactic acid, creatinine, l-alanine, glycine, and l-cysteine levels, and amino acids metabolism pathways associated with glycolysis were significantly altered, including alanine, aspartate, and glutamate metabolism; glycine, serine, and threonine metabolism, which were involved in the regulation of blood pressure. Integrated multi-omics analysis revealed that changes in *Paraprevotella*, *Erysipelotrichaceae,* and genera from *Clostridiales* are associated with metabolic disorders. Gene functional predication analysis based on 16S Ribosomal RNA sequences showed that the dysfunction in hepatic metabolism were correlated with enhanced lipopolysaccharide (LPS) biosynthesis and apoptosis in gut microbes. Curcumin (50 mg/kg/d) might reduce gut microbes-associated LPS biosynthesis and apoptosis, partially reverse metabolic dysfunction, ameliorate renal oxidative stress, and protect against salt-sensitive hypertension.

## 1. Introduction

Growing evidence suggests that salt-sensitive hypertension is an independent risk factor for cardiovascular disease, accounting for nearly 50% of all patients with hypertension and 25% of the healthy population [1]. High salt consumption plays a critical role in the development of salt-sensitive hypertension. The recommended daily salt intake is no more than 5 g by the World Health Organization [2]. However, daily salt consumption exceeds the recommended levels in most regions [3], which poses a threat to the prognosis of patients with salt-sensitive hypertension. Kidneys play a critical role in the modulation of blood pressure (BP) by regulation of the sodium absorption and its excretion via the renal sodium transporters [4,5]. Abundant evidence indicates that renal metabolic dysfunctions, such as carbohydrate and lipid metabolism disorders caused by a high-salt diet (HSD), are closely related to the onset of hypertension [6]. However, the effects of high salt consumption are systemic, and the impact of metabolic dysfunction of extra-renal organs upon the development of hypertension needs to be further explored.

A large number of gut microbiomes presented in the cecum play a crucial role in ingesting nutrients from the diet, with some studies demonstrating the association between gut microbiomes and host metabolism [7,8]. Lipopolysaccharide (LPS), amino acids, and short chain fatty acid mediates crosstalk between gut microbes and host metabolism [9]. However, the involvement of the gut microbiota in the regulation of salt-sensitive hypertension requires further confirmation. Moreover, experimental evidence demonstrates that high salt intake directly changes the gut microbial composition followed by the changes in metabolites, and 80% of the blood supply in the liver is received from the intestine through the portal vein; thus, the liver becomes the “first-hit” organ for HSD feeding and gut microbial metabolites [10,11]. For instance, high salt intake is independently associated with an increased risk of nonalcoholic fatty liver disease (NAFLD) and advanced liver fibrosis [12]. Therefore, dietary sodium can affect the gut microbial composition, and disturb hepatic metabolism; however, the causal relationship remains unclear. Omics analysis is becoming an effective approach to study metabolic diseases. Metabolomics analysis focuses on the small molecular metabolites, which are the downstream products of gene expression. Metabolomics analysis is not only useful for the identification of biology biomarker but also helpful in the disclose of specific metabolic pathways in disease process [13].

The Dahl salt-sensitive rat strain SS/JrHsd/McwiCrl (denoted SS) is typically used as a salt-sensitive hypertensive rat model [14]. Our previous study suggested that HSD feeding changes the metabolic pathways in the kidneys of SS rats, including amino acid metabolism and carbohydrate metabolism [15]. However, it needs to be confirmed whether HSD feeding would induce similar changes in hepatic metabolism.

Curcumin is an extract from turmeric (*Zingiberaceae*), and has long been used as an anti-inflammatory drug [16]. In addition, the beneficial effects of curcumin in the treatment of chronic diseases such as diabetes, metabolic syndrome, gut inflammation, liver injury, and cirrhosis as well as modulating signaling pathway have been stated [17,18,19]. However, the effect of curcumin on salt-sensitive hypertension of SS rats is unclear. In this study, an untargeted metabolomics approach was used to analyze the changes in the metabolic processes of liver tissues, 16s rRNA sequencing was performed to identify the changes in gut microbiome after HSD feeding, and multi-omics integration analysis was performed to reveal the relationship between hepatic metabolites and gut microbial composition. In addition, curcumin was used to protect against salt-induced hypertension. This study comprehensively analyzed the contribution of multiple organs to salt-sensitive hypertension, and explored the underlying mechanism of curcumin in the treatment of salt-sensitive hypertension, which will provide a scientific basis for metabolic management of salt-sensitive hypertension.

## 2. Materials and Methods

### 2.1. Ethical Statement

This experiment was performed in accordance with the guidelines of the Care and Use of Laboratory Animals of the National Institutes of Health, and the animal experimental procedures were approved by the Biomedical Ethics Committee of Xi’an Jiaotong University (Xi’an, China) [Approval No.:XJTUSLST-2017-02].

### 2.2. Treatment and Sample Collection

Eight-week-old male Dahl salt-sensitive rat strain SS/JrHsd/McwiCrl (denoted SS) rats (220–250 g) were obtained from Vital River Laboratory (Beijing, China). Rats were housed at a temperature of 25 °C with a daily 12 h light/dark cycle. Twenty-one SS rats were randomly divided into three groups (*n* = 7 per group). The rats were fed a normal-salt AIN-76A diet (NS, 0.4% NaCl), high-salt AIN-76A diet (HS, 8% NaCl), or high-salt AIN-76A diet with curcumin (HSCUR, 50 mg/kg/day) by intragastric gavage (ig). The diet was obtained commercially from Keao Xieli Co., Ltd. (Beijing, China). Curcumin was obtained from Puripharm Co., Ltd. (Huzhou, China). Rats in the NS and HS groups were administered distilled water as a substituent for curcumin, through ig. Rats from each group were housed in three different cages (2 or 3 rats in per cage). The food and water intake, and body weight were measured daily at 8:00 a.m., and the mean of the three cages was calculated to obtain the daily food and water intake. After 5 weeks, overnight-fasted rats were sacrificed after anesthetization. The livers, kidneys, and hearts were removed after saline infusion. The experimental samples were stored at −80 °C for further analysis.

### 2.3. Blood Pressure (BP) Measurement

The acclimatization training for BP measurement was performed one week before the formal experiment. BP was measured using tail-cuff plethysmography with a CODA-4 computerized system (Kent Scientific Corporation, Torrington, CT, USA) at 2:00 p.m. twice a week for five weeks.

### 2.4. Metabolomics Analysis of the Liver and Kidney Tissues

Metabolomic analysis of the liver and kidney tissues was performed as previously described, with some modification [20]. Briefly, the liver and kidney tissues were extracted by chloroform/methanol/water (2:5:2, volume ratio) mixture with an extraction ratio (1:10, weight/volume), d27-myristic acid (3 mg/mL, Sigma-Aldrich, Saint Louis, MO, USA) was added to the extraction buffer as an internal standard. A total of 200 μL of supernatant was transferred to a new vial for freeze-drying and concentration. A pooled quality control (QC) sample was prepared by taken 30 µL of supernatant from each sample, and similarly 200 µL of mixed extract were concentrated in the same way with sample treatment. The concentrates were derivatized by 100 μL of methoxyamine for 60 min at 37 °C, and 100 μL of N-methyl-N-trimethylsilyltrifluoroacetamide (MSTFA) with 1% trimethylchlorosilane (TMCS, Sigma-Aldrich, Saint Louis, MO, USA) for 60 min at 60 °C. About 1 μL of derivative was used for the detection of metabolites. The metabolomics data were acquired using gas chromatography tandem mass spectrometer (GC–MS, Agilent 7890A-5975C, Wilmington, DE, USA). A DB–5 column (30 m × 0.25 mm i.d., 0.5 μm in film thickness, Agilent J&W Scientific, USA) was used for chromatographic separation, and the data were acquired using the Agilent retention time locking (RTL) system. d27-myristic acid was used as internal standard for retention time locking. Helium was used as the carrier gas at a rate of 1.0 mL/min. Column temperature was raised from 60 °C to 300 °C at a rate of 10 °C/min. The raw data acquired from GC–MS were imported to automated mass spectral deconvolution software (AMDIS, National Institute of Standards and Technology, US) for peak detections by comparison of the m/z and the retention time with the Agilent FiehnLib mass spectral library [21]. The minimum match factor in the AMDIS was set to 70. The peak alignment was performed by MATLAB software. The compounds were filtered, and the compounds with a prevalence of less than 80% in all samples were discarded. Finally, the peak area of each compound was normalized using the total peak area to obtain the relative intensity of each compound. Principal component analysis (PCA) and orthogonal partial least squares discriminant analysis (OPLS–DA) was performed using SIMCA (v14.1). The differential compounds were screened using the non-parametric Mann–Whitney–Wilcoxon test with a false discovery rate (FDR) control using Benjamini and Hochberg (BH) method. The threshold of the adjusted *p*-values was set as no more than 0.05. The pathway enrichment analysis based on the differential metabolites was performed using the Metaboanalyst online tool [22]. Pearson’s correlation coefficient (r) of hepatic metabolites was calculated using SPSS 20 (IBM, Armonk, NY, USA), and the threshold of absolute r value was set as more than 0.5 with a *p*-value less than 0.05. The correlation network based on r values was constructed using Gephi (v0.9.2). The centralities of metabolites in the correlation network were evaluated using closeness centrality, betweenness centrality, and eigenvector centrality. A three-dimensional scatter plot of the centralities was plotted using OriginPro (v9.1).

### 2.5. 16S Ribosomal RNA Sequencing

Total DNA was extracted from fecal samples using a Metagenomic DNA Isolation Kit (MoBio, Carlsbad, CA, USA). The V4 region of 16S ribosomal DNA was amplified using the forward primer 515F and the reverse primer 806R. Sequencing was performed on an Illumina NovaSeq 6000 platform at Guhe Info Technology Co., Ltd. (Hangzhou, China), and the raw data were analyzed using Quantitative Insights Into Microbial Ecology (QIIME, 1.9.0) [23]. Analyses of beta-diversity and species enrichment were performed as previously described [24]. Briefly, PCA based on weighted UniFrac distance was performed to determine the differences in operational taxonomic unit (OTU) composition among experimental groups. Linear discriminant analysis (LDA) effect size (LEfSe) and random forestry (RF) models were performed to explore the differential microbes among groups and to discriminate the samples from different groups. A cladogram of LEfSe analysis was employed to present the variations in spatiotemporal distribution. Only taxa with a LDA score (log 10) greater than 2 at a *p*-value less than 0.05 were considered differential gut microbes. Phylogenetic Investigation of Communities by Reconstruction of Unobserved States (PICRUSt) was used for functional prediction analysis based on microbial genomics [25].

### 2.6. Omics Dataset Pretreatment

The integration analysis of the gut microbial dataset and hepatic metabolomics dataset was conducted using the MetaboAnalyst online tool [22]. Briefly, differential analysis was performed using a one-way ANOVA test, and the difference threshold was set at a FDR adjusted (using the BH method) *p*-value less than 0.05. Normalization (by median), log transformation (base 10), and dataset scaling (mean centering) were conducted before integration. Spearman’s correlation coefficient (ρ) threshold of the similarity matrix was set as no less than 0.5 at a *p*-value less than 0.05.

### 2.7. Biochemical Analysis

The activities of glucose-6-phosphate dehydrogenase (G6PD) and 6-phosphogluconate (6PGD) in the renal tissues were examined using assay kits obtained from Solarbio Co., Ltd. (Beijing, China). The levels of hydrogen peroxide (H_2_O_2_), malonaldehyde (MDA), oxidized glutathione (GSSG), reduced glutathione (GSH) levels, and the activities of catalase (CAT), superoxide dismutase (SOD), glutathione reductase (GR), and glutathione S-transferase (GST) in renal tissues were measured using assay kits obtained from Nanjing Jiancheng Institute of Biotechnology (Nanjing, China).

### 2.8. Statistical Analysis

The biochemical parameters were presented as mean ± standard deviation (SD), and were compared by one-way ANOVAs followed by the Tukey HSD post hoc test among the three groups, *p*-value less than 0.05 was considered significant.

## 3. Results

### 3.1. Characteristics of SS Rats after 5 Weeks of Treatment

No significant differences were detected in body, kidney, and liver weights among the NS (normal salt-diet), HS (high salt-diet), and HSCUR (high salt-diet supplemented with curcumin) groups. However, the heart weight was slightly lower in the HSCUR group than in the HS group (*p* < 0.05). The slightly lower body weight and heart weight in HSCUR group compared to HS group might be related to the weight loss and heart protective effects of curcumin [26,27]. Food intake increased significantly in SS rats fed a HSD diet compared to those fed a normal-salt diet (NSD, *p* < 0.01), while curcumin intervention had no significant effect on food intake. Notably, HSD feeding induced a marked increase in daily water intake (*p* < 0.0001), and curcumin administration reduced water intake effectively as compared to the HS group (*p* < 0.01, Table 1).

### 3.2. Curcumin Alleviated Salt-Induced Hypertension in SS Rats

**Figure 1:**
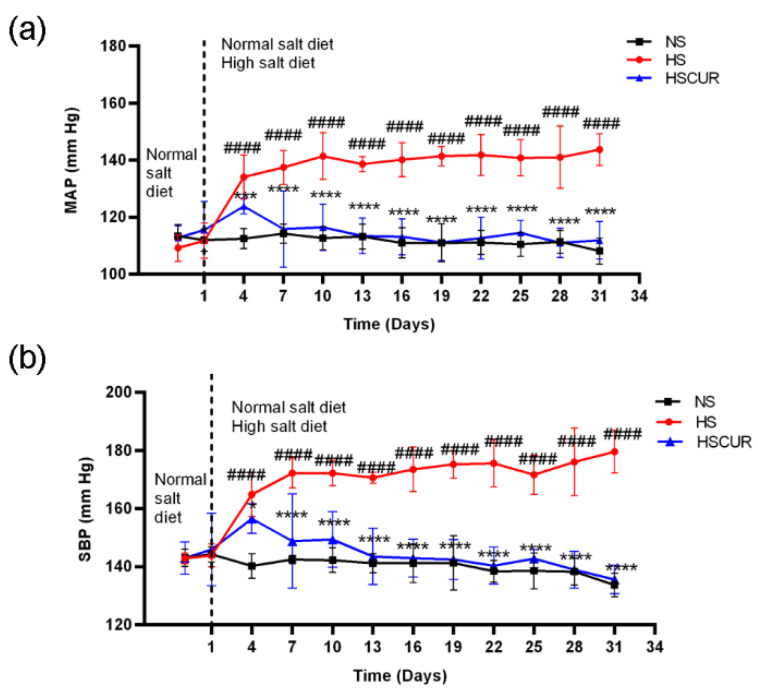
Curcumin reverted the high-salt diet (HSD)-induced increase in blood pressure. Changes in mean arterial pressure (**a**), and systolic blood pressure (**b**) among normal salt (NS), high salt (HS), and HS diet with curcumin (HSCUR) rats. Note: data are shown as the mean ± SD (*n* = 7 per group), ^####^ *p* < 0.0001 denotes the significant levels of difference between the NS and HS groups; **** *p* < 0.0001, *** *p* < 0.001, and * *p* < 0.05 denote the significant levels of difference between the HS and HSCUR groups.

The effects of curcumin on blood pressure (BP) were assessed in this study. The mean arterial pressure (MAP, Figure 1a) and systolic blood pressure (SBP, Figure 1b) increased significantly in SS rats after 4 days of HSD feeding by about 20 mm Hg, whereas curcumin intervention effectively inhibited the HSD-induced increase in MAP and SBP by 10 mm Hg. The antihypertensive effects of curcumin exerted from the fourth day of high salt intake. On the seventh day, the MAP increased to about 140 mm Hg, and the SBP increased to about 170 mm Hg in the HS group, and curcumin intervention could inhibit the increase in MAP and SBP by approximately 20 mm Hg.

### 3.3. Curcumin Administration Restored the Salt-Induced Variations in Hepatic Metabolism

**Figure 2:**
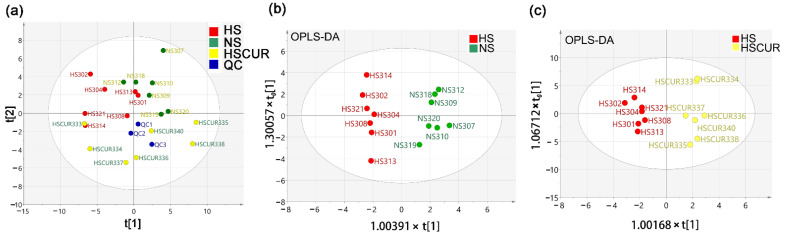
Hepatic metabolomics analysis. Score plot of principal component analysis (PCA) (**a**) and orthogonal partial least squares discriminant analysis (OPLS-DA) between NS and HS (**b**), or HS and HSCUR groups (**c**).

Previous studies have suggested that HSD feeding could induce metabolic disorders, which is related to the onset of hepatic disease [28,29]. Herein, an untargeted metabolomics approach was used to analyze the alterations in hepatic metabolism among the NS, HS, and HSCUR rats. First, PCA was applied for an overview of the global metabolic pattern. The OPLS-DA was performed to discriminate different experimental groups. Score plots of PCA (Figure 2a) showed an obvious separation among the different groups, suggesting that HSD feeding and curcumin administration significantly changed the hepatic metabolic pattern of SS rats. No outlier was shown in the PCA score plot; thus, all the experimental samples were used for further analysis. The score plot of the OPLS-DA model showed a complete separation between NS and HS groups, with a R2Y value of 0.9605 and a Q2 value of 0.6877 (Figure 2b). Moreover, Figure 2c showed a good separation between HS and HSCUR groups with a R2Y value of 0.9599 and a Q2 value of 0.8513. Higher R2Y values and Q2 values exhibited acceptable discriminating abilities of these OPLS-DA models.

**Figure 3:**
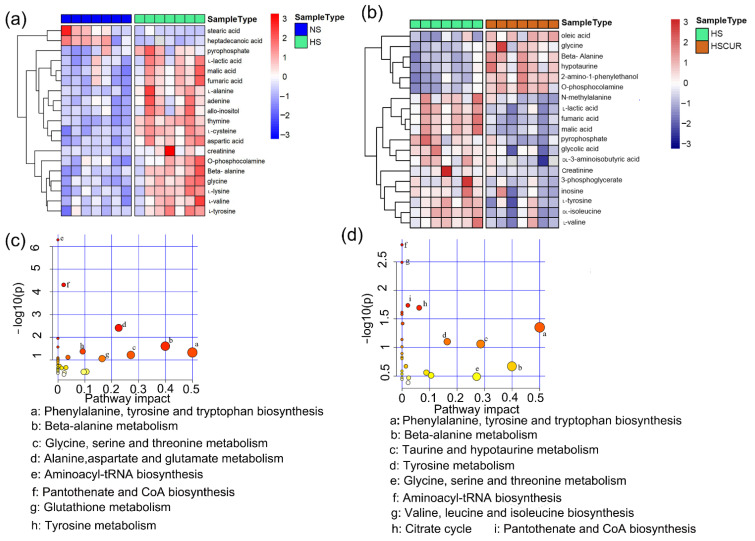
Hepatic metabolomics analysis. Differential metabolites in liver tissues between the HS and NS groups (**a**), or the HS and HSCUR groups (**b**); metabolic pathway enrichment analysis based on differential metabolites between the NS and HS groups (**c**), or the HS and HSCUR groups (**d**). The difference in the Mann–Whitney–Wilcoxon test with the false discovery rate (FDR) using Benjamini–Hochberg (BH) method adjusted *p*-value less than 0.05 was considered significant. Colors in sub-figures (**c**,**d**) were determined by –log10 (*p*) values, pathways with higher –log10 (*p*) were shown in red, and vice were shown in yellow.

Mann–Whitney–Wilcoxon test with FDR adjustment by BH method was applied to screen the differential metabolites. Metabolites with adjusted *p*-value less than 0.05 were considered differential metabolites. Differential metabolites in the liver tissues between the NS and HS groups were shown in Figure 3a. In the heatmap, the horizontal axis represents sample category, and the vertical axis represents differential compounds. The closer to red in the heatmap denoted the higher relative abundance of the compound. Nineteen metabolites were significantly altered, among which l-lactic acid, malic acid, fumaric acid, l-alanine, adenine, allo-inositol, thymine, l-cysteine, aspartic acid, creatinine, O-phosphocolamine, beta-alanine, glycine, l-lysine, l-valine, and l-tyrosine were significantly enriched in the HS groups. Perturbations in phenylalanine, tyrosine, and tryptophan biosynthesis; beta-alanine metabolism; glycine, serine, and threonine metabolism; alanine, aspartate, and glutamate metabolism; tyrosine metabolism, and glutathione metabolism were detected after HSD feeding (Figure 3c). Expectedly, curcumin administration modulated the salt-induced changes in hepatic metabolism. For instance, the relative abundances of N-methylalanine, l-lactic acid, fumaric acid, malic acid, pyrophosphate, creatinine, l-tyrosine, and l-valine were markedly reduced in the HSCUR group compared to HS group, among which l-lactic acid, fumaric acid, malic acid, creatinine, l-tyrosine, and l-valine were significantly enriched due to HSD when compared to NS group. However, the abundance of 2-amino-1-phenylethanol, beta-alanine, O-phosphocolamine, hypotaurine, glycine, and oleic acid levels were higher in the HSCUR than in the HS group (Figure 3b). In addition, phenylalanine, tyrosine, and tryptophan biosynthesis; beta-alanine metabolism; taurine and hypotaurine metabolism; tyrosine metabolism; glycine, serine, and threonine metabolism and the citrate cycle (TCA cycle) were significantly altered in HSCUR group compared to HS group (Figure 3d). Overall, curcumin intervention partially reduced salt-induced accumulation of metabolites.

**Figure 4:**
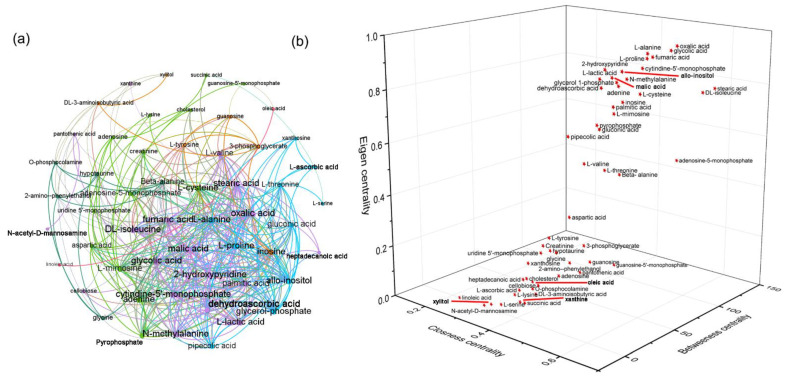
Hepatic metabolomics analysis. Metabolic correlation network analysis (**a**), and three-dimensional scatter plot of centrality indices of the correlation network (**b**). Pearson’s correlation coefficient (r) was used to construct the metabolic correlation network (using Gephi-0.9.2), and the threshold of the absolute correlation coefficient was set as no less than 0.5 at *p*-value less than 0.05.

Next, metabolic correlation analysis was used to identify the key metabolites that play pivotal roles in maintaining metabolic homeostasis, and the changes in the key metabolites suggested an overall disruption in metabolic homeostasis. A metabolic correlation network was constructed based on Pearson’s correlation coefficient (r) among the metabolites, with metabolites as the nodes and the correlation coefficient as the edges. In the correlation network, the node size was set according to the degree of centrality, and the weights of the edges were set according to the r values. Oxalic acid, glycolic acid, l-alanine, l-proline, fumaric acid, 2-hydroxypyridine, l-lactic acid, and N-methylalanine had higher degrees in this network, with relatively large nodes, suggesting that these compounds have higher connectivity with other metabolites (Figure 4a). In addition, the centralities of the metabolites in this network were evaluated using the eigenvector centrality, closeness centrality, and betweenness centrality. Oxalic acid, glycolic acid, l-alanine, l-proline, l-lactic acid, and fumaric acid were found with higher centralities, indicating critical positions in maintaining the metabolic homeostasis (Figure 4b). Changes in the relative abundance of compounds, such as l-alanine, fumaric acid and l-lactic acid, which were at central positions in the network, would cause a serious impact on the overall metabolic network.

### 3.4. HSD and Curcumin Administration Changed the Gut Microbial Community and Functional Potential

**Figure 5:**
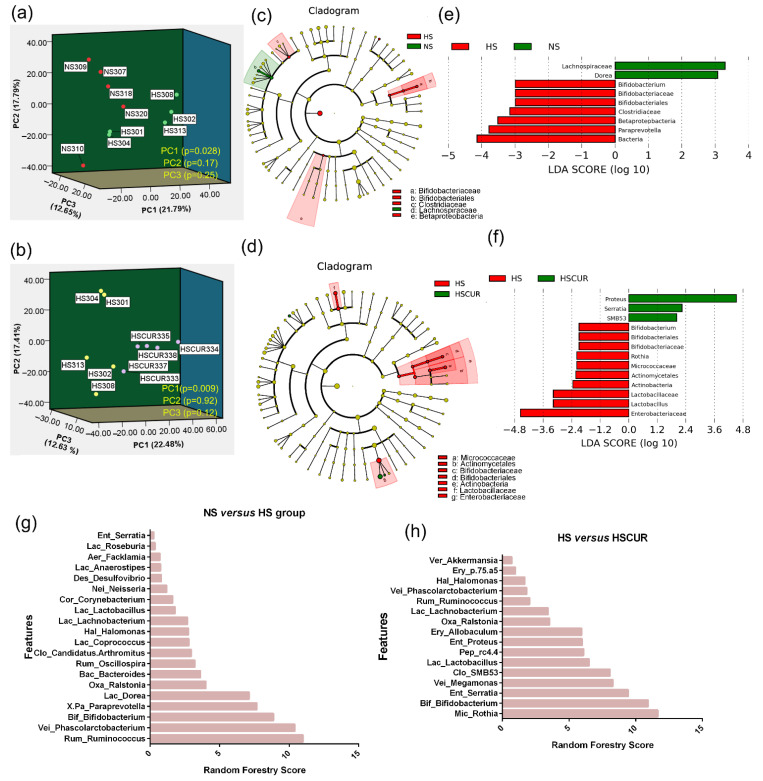
HSD feeding and curcumin administration changed the gut microbial composition in SS rats. Score plot of PCA analysis based on weighted UniFrac distance between the NS and HS groups (**a**), or the HS and HSCUR groups (**b**); the cladogram of linear discriminant analysis (LDA) effect size (LEfSe) analysis between the NS (green) and HS (red) groups (**c**), and LDA scores of differential microbial communities between the NS and HS groups (**d**); the cladogram of the LEfSe analysis between the HS (red) and HSCUR (green) groups (**e**), and the LDA scores of differential microbial communities between the HS and HSCUR groups (**f**); importance level of microbial communities for the classification between the HS and NS groups (**g**), or HSCUR (**h**) groups based on random forestry models.

As described above, significant changes in organic and amino acids were observed in the liver tissues of SS rats after HSD feeding. Given that the livers are “first-hit” organs for exposure to microbial metabolites via the portal vein, and changes in gut microbial compositions are often followed by a disturbance in the organic acid metabolism of the host [30,31], thus, 16S ribosomal RNA sequencing was performed to report the gut microbial alterations after HSD and curcumin administration in this study. First, the influence of HSD and curcumin on the beta diversity of the gut microbiota was assessed using PCA analysis. The PCA plots based on weighted UniFrac distance demonstrated a significantly differential distribution in the first principal component (PC1) between the HS and NS (*p*-value = 0.028; Figure 5a), or HS and HSCUR groups (*p*-value = 0.009; Figure 5b), suggesting that fecal samples of HS and HSCUR groups exhibited difference in beta diversity within microbial communities. Linear discriminant analysis (LDA) effect size (LEfSe) was performed to identify biomarker taxa in the gut microbiota after the experimental treatment. The cladogram demonstrated the structure of the fecal microbiota and the predominant bacteria in the NS and HS groups (Figure 5c). HS rats display different microbiome profiles from NS rats. The score plot of LDA showed nine differential taxonomic clades between NS and HS rats (LDA score > 3, *p* < 0.05). Specifically, the marked loss in *Lachnospiraceae* and *Dorea*, and the enrichment of *Paraprevotella*, *Betaproteobacteria, Clostridiaceae*, and *Bifidobacterium* were detected in HS rats compared to NS rats (Figure 5e). A decrease in *Lachnospiraceae* abundance has been identified as a microbial biomarker associated with chronic kidney disease [32]. The differences in the microbiome profiles between the HS and HSCUR groups were shown in Figure 5d. Enrichment of the genera *Proteus*, *Serratia*, and *SMB53* was observed, whereas marked loss in *Enterobacteriaceae*, *Lactobacillus*, *Actinobacteria*, *Actinomycetales*, *Micrococcaceae*, *Rothia*, and *Bifidobacterium* was detected in HSCUR rats as compared to HS rats (LDA score > 2, *p* < 0.05, Figure 5f). The random forestry (RF) model assisted us in further identifying the differences in the microbial composition. The results of the RF model showed that *Rum_Ruminococcus*, *Vei_Phascolarctobacterium*, *Bif_Bifidobacterium*, *X.Pa_Paraprevotella*, *Lac_Dorea*, *Oxa_Ralstonia*, *Bac_Bacteroides*, *Rum_Oscillospira*, *Clo_Candidatus.Arthromitus*, *Lac_Coprococcus*, *Hal_Halomonas*, *Lac_Lachnobacterium*, and *Lac_Lactobacillus*, etc., played critical roles in the classification between the HS and NS groups at the genus level (Figure 5g). The microbial taxa with greater importance in the discrimination of the HS and HSCUR groups included *Mic_Rothia*, *Bif_Bifidobacterium*, *Ent_Serratia*, *Vei_Megamonas*, *Clo_SMB53*, *Lac_Lactobacillus*, *Pep_rc4.4*, *Ent_Proteus*, *Ery_Allobaculum*, *Oxa_Ralstonia*, *Lac_Lachnobacterium*, and *Rum_Ruminococcus,* etc., at the genus level (Figure 5h). Overall HSD altered gut microbial composition in a manner that favors colonization of *Bifidobacterium, Paraprevotella, clostridiaceae* and other bacteria, whereas curcumin supplementation could reduce the abundance of *Enterobacteriaceae,* lactic acid-producing bacteria *(Bifidobacterium* and *Lactobacillus),* and *Mic_Rothia*.

### 3.5. HSD and Curcumin Administration Influences Hepatic Metabolism by Changing the Gut Microbial Composition

**Figure 6:**
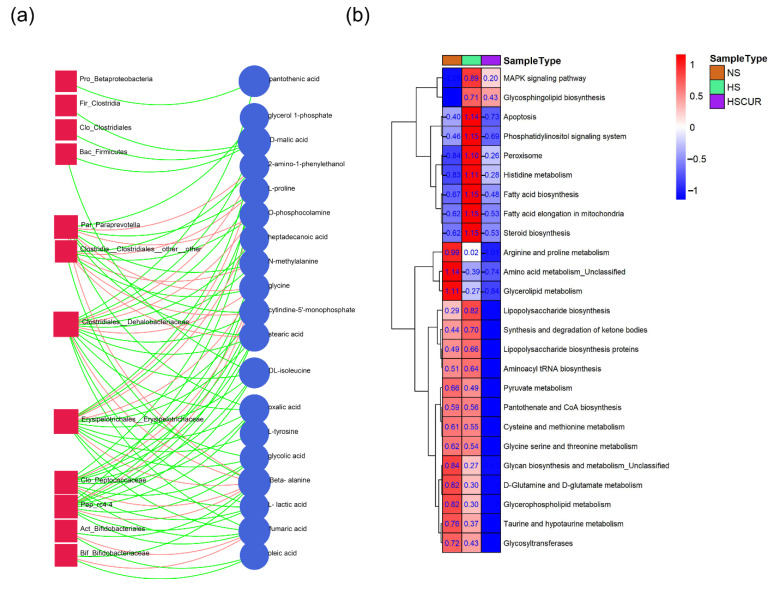
Integrated analysis of gut microbiota dataset and hepatic metabolomics dataset, and functional enrichment analysis based on microbial genomics. Integrated analysis of gut microbiota dataset and hepatic metabolomics dataset (**a**), functional enrichment analysis based on gut microbial genomics (**b**). The red squares represent the key differential microbiota, the blue circles represent the key differential hepatic metabolites, the red lines denote a positive correlation, and the green lines denote a negative correlation between microbial species and hepatic metabolites in figure (**a**). In figure (**b**), the red color represents high abundance and the blue color denotes low abundance, the numbers (scaled) in the block denote the relative abundance, Kruskal–Wallis test was used for screening the differential function pathways, and those pathways with raw *p*-values less than 0.05 and a FDR adjusted *p*-value lower than 0.1 were considered significant.

The integrated analysis of hepatic metabolomics and gut microbiota provided a comprehensive understanding of the metabolic regulatory mechanism in the liver-gut axis. In this study, integration analysis was employed to identify key features of hepatic metabolites or microbial communities that are closely related to each other. In the integrated correlation network, the node size was set according to the degree of key features in the correlation network; red edges denoted positive relationships, and green edges represented the negative relationship between metabolites and microbial communities. The results demonstrated that *Par_Paraprevotella*, *Clo_Clostridiales_other_other*, *Clo_Dehalobacteriaceae*, *Clo_Peptococcaceae*, *Pep_rc4-4*, and *Ery_Erysipelotrichaceae* were prominent nodes in the network, which were closely related to hepatic metabolites, including beta-alanine, l-lactic acid, malic acid, fumaric acid, l-tyrosine, and o-phosphocalamine (Figure 6a). Beta-alanine, fumaric acid, malic acid, and l-lactic acid, which were upregulated in HS rats, were also prominent nodes in this network (Figure 6a). It seemed that *Clostridiales* and *Erysipelotrichaceae* played a dominant role in the perturbation of hepatic metabolism. PICRUSt was employed to predict the microbial function based on 16S rRNA sequencing data. PICRUSt analysis revealed 104 function pathways in level 3 with significant differences between NS, HS, and HSCUR rats (Table S1, Kruskal–Wallis raw *p*-value less than 0.05, FDR adjusted *p*-value less than 0.1). Notably, 25 pathways associated with metabolism, signaling, and REDOX balance are shown in Figure 6b. MAPK signaling pathway, glycosphingolipid biosynthesis, apoptosis, phosphatidylinositol signaling system, peroxisome, histidine metabolism, fatty acid biosynthesis, fatty acid elongation in mitochondria, steroid biosynthesis, lipopolysaccharide (LPS) biosynthesis, LPS biosynthesis proteins, synthesis and degradation of ketone bodies, and aminoacyl tRNA biosynthesis were significantly enriched in the HS group compared to NS group. However, amino acid metabolism pathways including arginine and proline metabolism, cysteine and methionine metabolism, d-glutamine and d-glutamate metabolism, glycine, serine, and threonine metabolism, and taurine and hypotaurine metabolism were markedly reduced in the HS group when compared to the NS group (Figure 6b). Curcumin intervention significantly inhibited the salt-induced enrichment in apoptosis, peroxisome, LPS biosynthesis, LPS biosynthesis proteins, and lipid biosynthesis (steroid biosynthesis and fatty acid biosynthesis) in gut microbes. Unexpectedly, the decrease in amino acids metabolism induced by HSD was not reversed by curcumin intervention; in contrast, curcumin significantly reduced microbial amino acids metabolism further (Figure 6b). This may be related to the decrease in the abundance of some microbial species caused by curcumin supplementation, which are shown in Figure 5f.

### 3.6. Curcumin Administration Reversed the Salt-Induced Changes in Renal Metabolism

**Figure 7:**
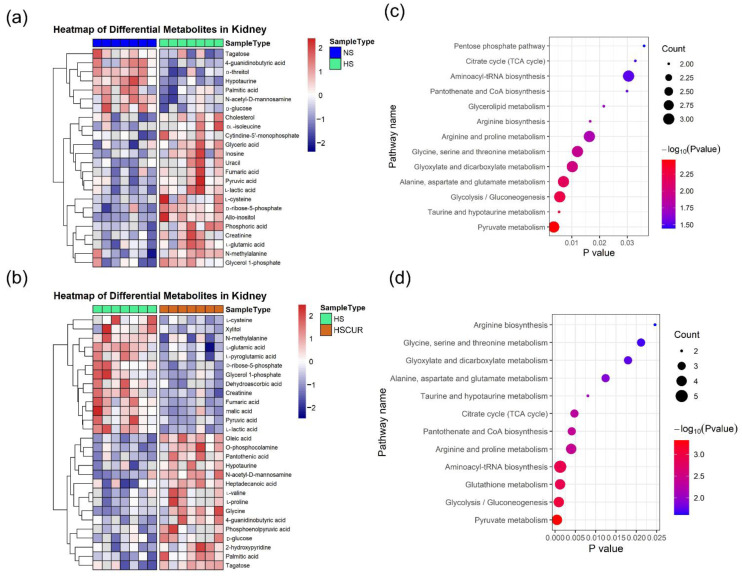
Statistical analysis of renal metabolomics dataset. Heatmap clustering analysis of differential metabolites between the NS and HS groups (**a**), the HS and HSCUR groups (**b**); pathway enrichment analysis based on differential metabolites between the NS and HS groups (**c**), the HS and HSCUR groups (**d**). The differential metabolites was identified according to Mann–Whitney–Wilcoxon test with the false discovery rate (FDR) adjusted *p*-value (<0.05).

Next, metabolomic analysis of renal tissues was performed to validate whether metabolic changes in the kidney coincided with those in the liver. A total of 24 metabolites were detected, with a significant difference between the NS and HS groups, among which 17 metabolites increased significantly in HS rats compared to NS rats (Figure 7a). Specifically, HSD intake induced an increase in the relative abundance of fumaric acid, l-lactic acid, l-cysteine, creatinine, pyruvic acid, l-glutamic acid, d-ribose-5-phosphate, and allo-inositol, etc., in the renal tissues, similar to the changes in the liver tissues; however, tagatose, 4-guanidinobutyric acid, hypotaurine, d-threitol, palmitic acid, and d-glucose decreased markedly in HS rats compared to the NS rats. These perturbed metabolic pathways based on these differential metabolites included pyruvate metabolism; taurine and hypotaurine metabolism; glycolysis/gluconeogenesis; alanine, aspartate, and glutamate metabolism; glyoxylate and dicarboxylate metabolism; glycine, serine, and threonine metabolism; arginine and proline metabolism; arginine biosynthesis; glycerolipid metabolism; the citrate cycle; and pentose phosphate pathway etc., (Figure 7c). Similarly, the main alterations in the renal metabolites after curcumin intervention were identified, including decreased levels of l-cysteine, xylitol, N-methylalanine, l-glutamic acid, d-ribose-5-phosphate, glycerol 1-phosphate, l-lactic acid, pyruvic acid, fumaric acid, d-malic acid, and creatinine, and increased levels of oleic acid, palmitic acid, d-glucose, tagatose, o-phosphocolamine, heptadecanoic acid, hypotaurine, l-proline, l-valine, and glycine, etc. (Figure 7b). The perturbed metabolic pathways after curcumin intervention in renal tissues included pyruvate metabolism; glycolysis/gluconeogenesis; glutathione metabolism; aminoacyl tRNA biosynthesis; arginine and proline metabolism; pantothenate and CoA biosynthesis; citrate cycle; taurine and hypotaurine metabolism; alanine, aspartate, and glutamate metabolism; glyoxylate and dicarboxylate metabolism; and glycine, serine, and threonine metabolism; and arginine biosynthesis (Figure 7d). Collectively, salt-induced increase in the levels of several key metabolites such as fumaric acid, l-lactic acid, creatinine, and l-cysteine was effectively reversed by curcumin.

**Figure 8:**
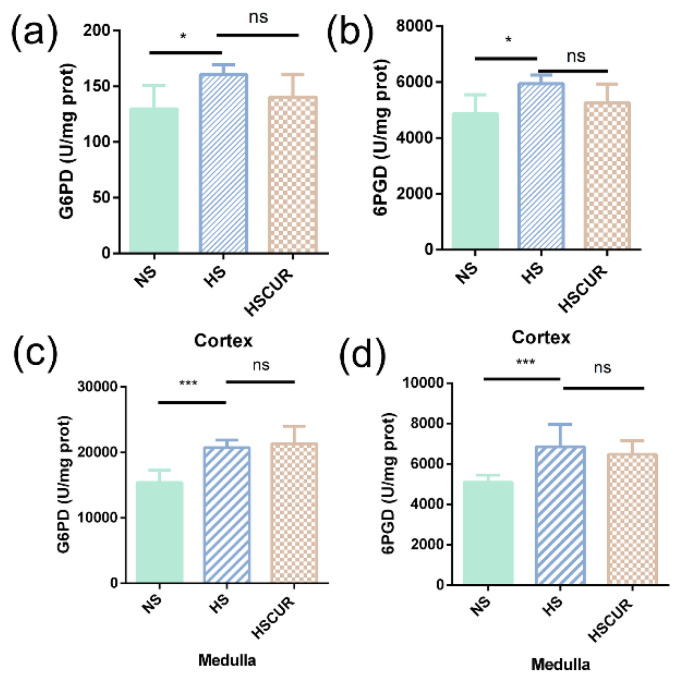
HSD induced a significant increase in glucose-6-phosphate dehydrogenase (G6PD) and 6-phosphogluconate dehydrogenase (6PGD) in the renal tissues. The activity of G6PD in the renal cortex (**a**) and medulla (**c**); the activity of 6PGD in the renal cortex (**b**) and medulla (**d**). ns denotes non significance, *** *p* < 0.001, * *p* < 0.05, and *p*-value was determined by one-way ANOVA test followed by Tukey’s test.

Based on our findings, the accumulation of l-lactic acid is a common salt-induced alteration in the liver and kidney. The aberrant citrate cycle and accumulation of l-lactic acid are commonly known as the Warburg effect, followed by activation of the pentose phosphate pathway (PPP) [33]. Therefore, the activities of G6PD and 6PGD, the key enzymes of the PPP, were detected in the renal tissues. As expected, the activities of G6PD and 6PGD increased significantly both in the renal cortex (*p* < 0.05, Figure 8a,b) and in the renal medulla (*p* < 0.001, Figure 8c,d) after HSD treatment; however, no significant changes were observed in the HSCUR group when compared to those in HS group. It seemed that curcumin could not effectively reverse the salt-induced activation of PPP in renal tissues.

**Figure 9:**
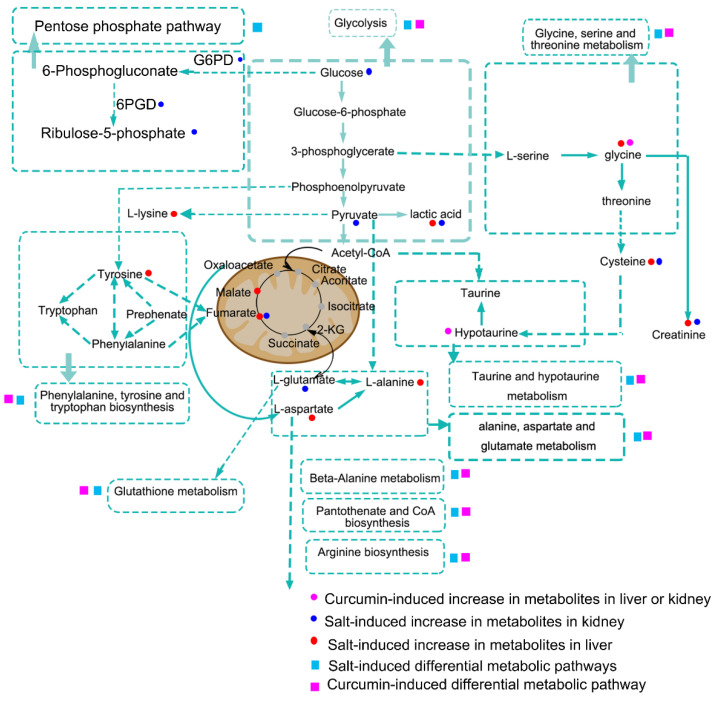
The schematic diagram of changes in the metabolic pathways after high salt intake in SS rats.

Collectively, metabolic changes in the liver and kidney after HSD and curcumin treatment were shown in Figure 9. Metabolic perturbations appeared to be closely related to the intermediate metabolites of glycolysis, which implies that HSD feeding might inhibit carbohydrate metabolism through the citrate cycle, and discontinuous flux in the citrate cycle promotes the enhancement of PPP and glycolysis. Subsequently, excessive amino acids and organic acids are derived from the intermediate metabolites of glycolysis, such as pyruvate, l-lactic acid, l-lysine, and l-alanine. Moreover, the increase in the levels of ribulose-5-phosphate, and the enzyme activities of G6PD and 6PGD suggest the activation of PPP in the kidney and further validates the above inference. In addition, amino acid metabolism pathways associated with glycolysis, including glycine, serine and threonine metabolism, phenylalanine, tyrosine and tryptophan biosynthesis, glutathione metabolism, taurine and hypotaurine metabolism, alanine, aspartate and glutamate metabolism, beta-alanine metabolism, and arginine biosynthesis, were disturbed by HSD. Incidentally, curcumin intervention also caused changes in those metabolic pathways. The therapeutic effect of curcumin was that it could reduce salt-induced abundance in pyruvate, l-lactic acid, alanine, fumaric acid, and creatinine, etc., in livers or in kidneys. In combination with the changes in microbial functions based on 16S rRNA sequencing data, we hypothesized that salt-induced changes in microbial composition promoted LPS synthesis, peroxisomes, and apoptosis, and these microbial-associated changes might further implicate in the regulation of host oxidative stress and metabolism.

### 3.7. Effect of Curcumin on REDOX Balance in SS Rats after HSD Feeding

**Figure 10:**
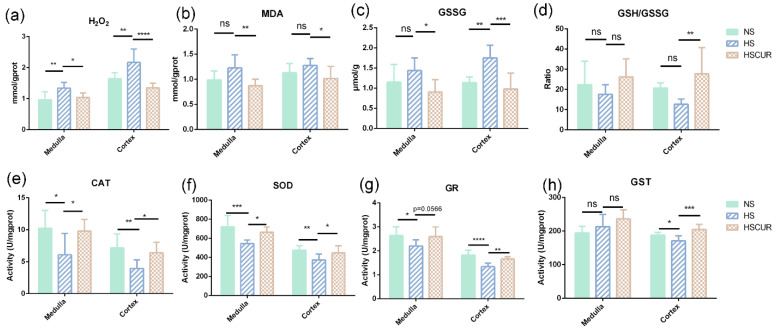
Effect of curcumin on REDOX balance in the kidney of SS rats after HSD feeding. Hydrogen peroxide (H_2_O_2_) levels (**a**), malonaldehyde (MDA) levels (**b**), oxidized glutathione (GSSG) levels (**c**), reduced glutathione/GSSG ratio (**d**), catalase activity (**e**), superoxide dismutase activity (**f**), glutathione reductase activity (**g**), and glutathione S-transferase activity (**h**). ns denotes non significance, **** *p* < 0.0001, *** *p* < 0.001, ** *p* < 0.01, * *p* < 0.05, and *p*-value was determined by one-way ANOVA test followed by Tukey’s test.

Metabolic disorders are commonly followed by mitochondrial antioxidant dysfunction and over expression of reactive oxygen species (ROS) [34,35]. Therefore, the renal levels of H_2_O_2_ and MDA were assessed. HSD feeding promoted a significant increase in renal H_2_O_2_ (*p* < 0.01), whereas curcumin intervention effectively inhibited the increase in H_2_O_2_ content in renal medulla (*p* < 0.05) and cortex (*p* < 0.0001) (Figure 10a). Moreover, the changing trend of MDA resembled that of H_2_O_2_ in the kidneys, while the difference was insignificant in the HS group compared to the NS group; however, curcumin intervention induced a significant reduction in MDA (*p* < 0.05, Figure 10b). A previous study reported that two GSH molecules donate one electron to GSSG under oxidative stress, which can be reduced back to GSH by the catalysis of GR [36]. Thus, the GSH/GSSG ratio, an indicator of the redox balance, was measured in this study. An increase in GSSG content was observed in the renal medulla (*p* > 0.05) and cortex (*p* < 0.01, Figure 10c), whereas curcumin inhibited the increase in GSSG in the renal medulla (*p* < 0.05) and cortex (*p* < 0.001, Figure 10c). The ratio of GSH/GSSG in the renal medulla and cortex was slightly reduced after HSD feeding (*p* > 0.05); however, curcumin intervention promoted a significant increase in the cortex (*p* < 0.01, Figure 10d).

The effects of HSD and curcumin intervention on renal antioxidant capacity were further assessed. The activities of catalase (CAT) and superoxide dismutase (SOD) in the kidney decreased markedly due to HSD, which were reversed by curcumin (Figure 10e,f). In addition, HSD feeding significantly reduced the activity of GR in the kidneys; however, curcumin intervention restored GR activity to some extent (Figure 10g). Glutathione S-transferase (GST) plays a critical role in protecting the cells against oxidative damage by accelerating the reaction between GSH and electrophilic xenobiotics [37]. GST downregulation was observed in the renal cortex of SS rats fed on HSD, and this decrease was reversed by curcumin intervention. However, no marked changes in GST were detected in the renal medulla (Figure 10h).

## 4. Discussion

This study validated that HSD feeding disturbs the hepatic metabolism of SS rats, which was partially consistent with the metabolic changes in the kidney and related to the changes in gut microbial composition. Taken together, this study suggested that salt-sensitive hypertension appears to be associated with alterations in metabolic processes, including glycolysis, the PPP, REDOX, and amino acids metabolism, which are derived from products of the intermediate metabolites of glycolysis (Figure 9). Furthermore, curcumin intervention protected SS rats against salt-induced hypertension, which is related to the restoration of metabolic disorders and the reduction of oxidative stress.

A previous study suggested that high salt intake promotes hepatic damage, such as NAFLD [38], and a supplement with 4% NaCl (*w*/*v*) in drinking water changes the hepatic cord morphology in mice [39]. Salt-induced hepatic damage includes mitochondrial dysfunction, which is involved in the regulation of lipid and carbohydrate metabolism; thus, mitochondrial dysfunction results in a decrease in energy supply and an increase in the glycolysis pathway [40]. In this study, HSD feeding induced perturbations in phenylalanine, tyrosine, and tryptophan biosynthesis; beta-alanine metabolism; alanine, aspartate, and glutamate metabolism; and glycine, serine, and threonine metabolism in the livers, all of which are derived from glycolic products (Figure 9). In addition, accumulation of the intermediate metabolites of citrate cycle such as fumaric acid is caused by fumarase deficiency, leading to inadequate carbohydrate metabolism after high salt intake in SS rats [41].

However, how the metabolic changes described above are involved in BP regulation remains unclear. A previous study suggested that a higher intake of phenylalanine causes a 14% increase in diastolic BP [42]. Alanine, aspartate, and glutamate metabolism pathway has been identified as a potential biomarker of essential hypertension in humans [43], and can contribute to insulin resistance, which in turn increases sodium reabsorption and promotes the development of essential hypertension [44]. Moreover, glycine, serine, and threonine metabolism pathway, which implicated in the antioxidant process of cells by regulating glutathione biosynthesis and NADPH production [45], are disturbed significantly after HSD feeding. A previous study demonstrated that fumaric acid accumulation leads to excessive ROS production and the salt-sensitive response of BP in SS rats [46]. The accumulation of fumaric acid in liver tissues may contribute to salt sensitivity through the same mechanism. In addition, a trial in the reduction of the renal mass confirmed the elevation of glycine in the liver, which was involved in the increased hepatic glycine methylation for organic osmolyte generation [47], in turn promoting the development of hypertension. Collectively, the accumulation of l-lactic acid, fumaric acid, malic acid, l-valine, and l-alanine might be related to the inhibition of the citrate cycle and the activation of amino acids production due to the conversion of glycolic products after HSD feeding. The elevated hepatic amino acids are implicated in the regulation of body water content and the production of organic osmolyte [47], which could induce hypertension by promoting physiological water retention in the body.

Hepatic metabolism disorders are also caused by alterations in microbial composition [48]. PCA plots of beta diversity showed that HSD and curcumin intervention significantly altered gut microbial composition (Figure 5a,b). Specifically, HSD induced an increase in *Paraprevotella*, *Betaproteobacteria*, *Clostridiaceae*, and *Bifidobacterium* and reduced the abundance of *Lachnospiraceae.* Consistently, a comparative study confirmed the enrichment of *Paraprevotella* and reduction of *Lachnospiraceae* in spontaneously hypertensive heart failure rats compared to normotensive Wistar Kyoto (WKY) rats [49].

Enrichment of immunogenic commensals (*Clostridiaceae and Paraprevotella*) were also observed in patients with liver diseases, and the relative abundance of *Clostridiaceae* increased with the severity of NAFLD [50,51,52]. Integrated analysis of gut microbiota and hepatic metabolism demonstrated that *Paraprevotella*, *Erysipelotrichaceae*, and genera from *Clostridiales* (*Peptococcaceae, Dehalobacreriaceae*, *and others)* were closely related to various differential metabolites, such as beta-alanine, l-lactic acid, fumaric acid, and l-proline. Furthermore, *Erysipelotrichaceae*, a key feature in the integration analysis, also showed a significant reduction in spontaneously hypertensive rats compared to WKY rats [49]. Thus, changes in the gut microbial composition are not a mere consequence but also cause perturbations in metabolic pathways. 

Microbial function enrichment analysis showed enriched apoptosis, peroxisomes, LPS biosynthesis, fatty acid biosynthesis, steroid biosynthesis, and the diminution of amino acid metabolism pathways after HSD feeding (Figure 6b). Consistent with a previous study, enhanced fatty acid synthesis was detected in rats with chronic kidney disease [53]. Moreover, increased peroxisomes and LPS biosynthesis in gut micobiome of HS rats may contribute to oxidative stress in salt-sensitive hypertension, which subsequently promoted apoptosis. LPS can shift metabolism away from oxidative phosphorylation and evoke the production of ROS [54]. The enhanced phosphatidylinositol signaling system in gut microbes, as a core-signaling pathway, modulates cellular proliferation, cellular differentiation, apoptosis, and membrane trafficking [55]. Overall, these were consistent with our findings in HS rats that accumulation of l-lactic acid, pyruvic acid, l-lysine, l-alanine, and fumaric acid, and activation of PPP suggested disorders in the citrate cycle, carbohydrate metabolism, and amino acid metabolism (Figure 9). Collectively, the host metabolic changes were closely related to the gut microbial composition, and this metabolic regulation might be achieved by modulating the LPS production.

Abundant evidence suggests that curcumin manifests health benefits by alleviating mitochondrial dysfunction and endoplasmic reticulum stress [56]. Therefore, the effects of curcumin on metabolic processes were investigated in this study. As expected, curcumin intervention reversed salt-induced hypertension, and subsequently, the hepatic changes in the metabolism induced by HSD were effectively reversed by curcumin. For instance, metabolomic biomarkers of hypertension, such as l-tyrosine, l-valine, fumaric acid, creatinine, and l-lactic acid [57], were reduced by curcumin in the liver tissues. In contrast, glycine and hypotaurine, which could lower BP [15], increased significantly in HSCUR rats compared to HS rats. Moreover, evidence supports that curcumin can regulate host metabolism by modulating gut microbial composition. In this study, curcumin intervention reduced the relative abundance of *Enterobacteriaceae*, *Lactobacillus*, *Actinomycetales*, *Rothia*, and *Bifidobacterium,* all of which are closely related to the severity of liver disease [51,58]. The PICRUSt analysis revealed that curcumin would reduce microbial LPS biosynthesis, apoptosis, peroxisomes, and fatty acid biosynthesis. Microbial functions predicted to be attenuated in the presence of curcumin also included amino acid metabolism, which might be caused by the reduction of gut microbiota after curcumin intervention. However, this is only a prediction based on gene function, and the effect of curcumin on microbial metabolism needs to be further explored.

Similar changes were detected in the renal tissues of the SS rats. For instance, salt-induced accumulation of amino acids (l-cysteine, _DL_-isoleucine, l-glutamic acid, and N-methylalanine) and organic acids (fumaric acid, pyruvic acid, and l-lactic acid) were effectively reversed by curcumin, which is consistent with the concept that changes in renal oxygen metabolism lead to alterations in metabolic intermediaries, which in turn are involved in BP regulation [6]. Flux discontinuities in the citrate cycle, enhanced PPP and glycolysis are closely related to perturbation in amino acids such as taurine and hypotaurine metabolism, and glutathione metabolism in this study, which will interrupt the redox balance and result in salt-sensitive hypertension [15]. As expected, curcumin significantly attenuated salt-induced oxidative stress in renal tissues by increasing antioxidant activity (Figure 10).

## 5. Conclusions

Comprehensive analysis of metabolism in the liver, kidney, and gut microbiota revealed that HSD disturbs global metabolism, resulting in the accumulation of derivatives of glycolysis, amino acids, furmaric acid, and l-lactic acid. In addition, HSD feeding would enhance microbial LPS synthesis, apoptosis, and peroxisomes. Overall, salt-induced metabolic dysfunction in the liver and kidney might be related to the enhancement of oxidative stress, and microbial LPS biosynthesis, and then the dual effects of liver and kidney contribute to the development of salt-sensitive hypertension. Curcumin can restore salt-induced metabolic dysfunction in the liver and kidney, reduce salt-induced enhancement of microbial LPS synthesis, apoptosis and peroxisomes, reverse salt-induced renal oxidative stress, and protect against salt-sensitive hypertension. In future, modulation of gut microbial composition is worth trying to restore salt-induced metabolic dysfunction.

## Figures and Tables

**Table 1:** Characteristics of SS rats after 5 weeks of treatment.

Parameters	NS	HS	HSCUR
Body weight (g)	284.7 ± 26.3	275.1 ± 17.8	257.0 ± 11.3
Kidney weight (g)	1.29 ± 0.107	1.23 ± 0.138	1.36 ± 0.151
Heart weight (g)	1.11 ± 0.034	1.11 ± 0.04	0.986 ± 0.046 *
Liver weight (g)	7.99 ± 0.33	7.34 ± 0.16	7.60 ± 0.24
Daily food intake (g/day)	17.4 ± 0.525	23.1 ± 0.609 ^##^	21.8 ± 1.98
Daily water intake (mL/day)	27.6 ± 1.30	77.3 ± 2.30 ^####^	64.8 ± 3.43 **

Note: ^#^ denotes a significant difference between NS and HS groups, ^##^ *p* < 0.01, ^####^ *p* < 0.0001. * denotes a significant difference between HS and HSCUR groups, * *p* < 0.05, ** *p* < 0.01. NS, normal salt group; HS, high salt group; HSCUR, HS diet supplemented with curcumin group.

## Data Availability

All data is contained within the article or supplementary material, and all data in this article is available for publication.

## Supplementary Materials

The following supporting information can be downloaded at: https://www.mdpi.com/article/10.3390/metabo12111076/s1, Table S1: Microbial function analysis by Phylogenetic Investigation of Communities using the Reconstruction of Unobserved states (PICRUSt) based on 16S rRNA sequencing data.

## Abbreviations

NSD, normal salt-diet; HSD, high-salt diet; CUR, curcumin; ig, intragastric administration, BP, blood pressure; SS, Dahl salt-sensitive rat; PCA, principal component analysis; OPLS-DA, orthogonal partial least squares discriminant analysis; OTU, operational taxonomic unit; LDA, linear discriminant analysis; LEfSe, linear discriminant analysis effect size; RF, random forestry; PICRUSt, Phylogenetic Investigation of Communities by Reconstruction of Unobserved States; G6PD, glucose-6-phosphate dehydrogenase; 6PGD, 6-phosphogluconate; H_2_O_2_, hydrogen peroxide; MDA, malonaldehyde; GSSG, oxidized glutathione; GSH, reduced glutathione; CAT, catalase; SOD, superoxide dismutase; GR, glutathione reductase; GST, glutathione S-transferase; LPS, lipopolysaccharide, PPP, pentose phosphate pathway.

## References

[1] Elijovich F., Laffer C.L., Sahinoz M., Pitzer A., Ferguson J.F., Kirabo A. (2020). The gut microbiome, inflammation, and salt-sensitive hypertension. Curr. Hypertens. Rep..

[2] Härtl G. (2013). WHO issues new guidance on dietary salt and potassium. Cent. Eur. J. Public Health.

[3] Appel L.J., Frohlich E.D., Hall J.E., Pearson T.A., Sacco R.L., Seals D.R., Sacks F.M., Smith S.C., Vafiadis D.K., Van Horn L.V. (2011). The importance of population-wide sodium reduction as a means to prevent cardiovascular disease and stroke: A call to action from the American Heart Association. Circulation.

[4] Zheng X., Zhao X., Jin Y., Zhou L., Yang P., Ahmad H., Tian Z. (2021). High salt diet contributes to hypertension by weakening the medullary tricarboxylic acid cycle and antioxidant system in Dahl salt-sensitive rats. Biochimie.

[5] Frame A.A., Wainford R.D. (2017). Renal sodium handling and sodium sensitivity. Kidney Res. Clin. Pract..

[6] Tian Z., Liang M. (2021). Renal metabolism and hypertension. Nat. Commun..

[7] Turnbaugh P.J., Ley R.E., Mahowald M.A., Magrini V., Mardis E.R., Gordon J.I. (2006). An obesity-associated gut microbiome with increased capacity for energy harvest. Nature.

[8] Schreiner J., Weber M., Loeschke K. (1998). Sodium chloride transport of normal and dietary enlarged rat cecum in vitro. Digestion.

[9] de Vos W.M., Tilg H., Van Hul M., Cani P.D. (2022). Gut microbiome and health: Mechanistic insights. Gut.

[10] Zhang Z., Li M., Cui B., Chen X. (2022). Antibiotic disruption of the gut microbiota enhances the murine hepatic dysfunction associated with a high-salt diet. Front. Pharmacol..

[11] Gao B., Jeong W.I., Tian Z. (2008). Liver: An organ with predominant innate immunity. Hepatology.

[12] Huh J.H., Lee K.J., Lim J.S., Lee M.Y., Park H.J., Kim M.Y., Kim J.W., Chung C.H., Shin J.Y., Kim H.S. (2015). High dietary sodium intake assessed by estimated 24-h urinary sodium excretion is associated with NAFLD and hepatic fibrosis. PLoS ONE.

[13] Tavares G., Venturini G., Padilha K., Zatz R., Pereira A.C., Thadhani R.I., Rhee E.P., Titan S.M.O. (2018). 1,5-Anhydroglucitol predicts CKD progression in macroalbuminuric diabetic kidney disease: Results from non-targeted metabolomics. Metabolomics.

[14] Abais-Battad J.M., Alsheikh A.J., Pan X., Fehrenbach D.J., Dasinger J.H., Lund H., Roberts M.L., Kriegel A.J., Cowley A.W., Kidambi S. (2019). Dietary effects on Dahl salt-sensitive hypertension, renal damage, and the T lymphocyte transcriptome. Hypertension.

[15] Yang P., Zhou L., Chen M., Zeng L., Ouyang Y., Zheng X., Chen X., Yang Z., Tian Z. (2022). Supplementation of amino acids and organic acids prevents the increase in blood pressure induced by high salt in Dahl salt-sensitive rats. Food Funct..

[16] Daily J.W., Yang M., Park S. (2016). Efficacy of turmeric extracts and curcumin for alleviating the symptoms of joint arthritis: A systematic review and meta-analysis of randomized clinical trials. J. Med. Food.

[17] Nouri-Vaskeh M., Malek Mahdavi A., Afshan H., Alizadeh L., Zarei M. (2020). Effect of curcumin supplementation on disease severity in patients with liver cirrhosis: A randomized controlled trial. Phytother. Res..

[18] Mantzorou M., Pavlidou E., Vasios G., Tsagalioti E., Giaginis C. (2018). Effects of curcumin consumption on human chronic diseases: A narrative review of the most recent clinical data. Phytother. Res..

[19] Zia A., Farkhondeh T., Pourbagher-Shahri A.M., Samarghandian S. (2021). The role of curcumin in aging and senescence: Molecular mechanisms. Biomed. Pharmacother..

[20] Wang L., Hou E., Wang L., Wang Y., Yang L., Zheng X., Xie G., Sun Q., Liang M., Tian Z. (2015). Reconstruction and analysis of correlation networks based on GC-MS metabolomics data for young hypertensive men. Anal. Chim. Acta.

[21] Kind T., Wohlgemuth G., Lee D.Y., Lu Y., Palazoglu M., Shahbaz S., Fiehn O. (2009). FiehnLib: Mass spectral and retention index libraries for metabolomics based on quadrupole and time-of-flight gas chromatography/mass spectrometry. Anal. Chem..

[22] Chong J., Soufan O., Li C., Caraus I., Li S., Bourque G., Wishart D.S., Xia J. (2018). MetaboAnalyst 4.0: Towards more transparent and integrative metabolomics analysis. Nucleic Acids Res..

[23] Yu M., Jia H., Zhou C., Yang Y., Zhao Y., Yang M., Zou Z. (2017). Variations in gut microbiota and fecal metabolic phenotype associated with depression by 16S rRNA gene sequencing and LC/MS-based metabolomics. J. Pharm. Biomed. Anal..

[24] Hu S., Li A., Huang T., Lai J., Li J., Sublette M.E., Lu H., Lu Q., Du Y., Hu Z. (2019). Gut microbiota changes in patients with bipolar depression. Adv. Sci..

[25] Langille M.G., Zaneveld J., Caporaso J.G., McDonald D., Knights D., Reyes J.A., Clemente J.C., Burkepile D.E., Vega Thurber R.L., Knight R. (2013). Predictive functional profiling of microbial communities using 16S rRNA marker gene sequences. Nat. Biotechnol..

[26] Jamilian M., Foroozanfard F., Kavossian E., Aghadavod E., Shafabakhsh R., Hoseini A., Asemi Z. (2020). Effects of curcumin on body weight, glycemic control and serum lipids in women with polycystic ovary syndrome: A randomized, double-blind, placebo-controlled trial. Clin. Nutr. ESPEN.

[27] Meng Z., Yu X.H., Chen J., Li L., Li S. (2014). Curcumin attenuates cardiac fibrosis in spontaneously hypertensive rats through PPAR-γ activation. Acta Pharmacol. Sin..

[28] Chen X., Zhang Z., Cui B., Jiang A., Tao H., Cheng S., Liu Y. (2020). Combination of chronic alcohol consumption and high-salt intake elicits gut microbial alterations and liver steatosis in mice. J. Agric. Food Chem..

[29] Choi Y., Lee J.E., Chang Y., Kim M.K., Sung E., Shin H., Ryu S. (2016). Dietary sodium and potassium intake in relation to non-alcoholic fatty liver disease. Br. J. Nutr..

[30] Daneberga Z., Nakazawa-Miklasevica M., Berga-Svitina E., Murmane D., Isarova D., Cupane L., Masinska M., Nartisa I., Lazdane A., Miklasevics E. (2021). Urinary organic acids spectra in children with altered gut microbiota composition and autistic spectrum disorder. Nord. J. Psychiatry.

[31] Golonka R.M., Vijay-Kumar M. (2021). Atypical immunometabolism and metabolic reprogramming in liver cancer: Deciphering the role of gut microbiome. Adv. Cancer Res..

[32] Lun H., Yang W., Zhao S., Jiang M., Xu M., Liu F., Wang Y. (2019). Altered gut microbiota and microbial biomarkers associated with chronic kidney disease. Microbiologyopen.

[33] Lu J., Tan M., Cai Q. (2015). The Warburg effect in tumor progression: Mitochondrial oxidative metabolism as an anti-metastasis mechanism. Cancer Lett..

[34] Rius-Pérez S., Torres-Cuevas I., Millán I., Ortega Á.L., Pérez S. (2020). PGC-1α, Inflammation, and oxidative stress: An integrative view in metabolism. Oxid. Med. Cell. Longev..

[35] Reuter S., Gupta S.C., Chaturvedi M.M., Aggarwal B.B. (2010). Oxidative stress, inflammation, and cancer: How are they linked?. Free Radic. Biol. Med..

[36] Giustarini D., Dalle-Donne I., Milzani A., Fanti P., Rossi R. (2013). Analysis of GSH and GSSG after derivatization with N-ethylmaleimide. Nat. Protoc..

[37] Habashy W.S., Milfort M.C., Rekaya R., Aggrey S.E. (2019). Cellular antioxidant enzyme activity and biomarkers for oxidative stress are affected by heat stress. Int. J. Biometeorol..

[38] Gao P., You M., Li L., Zhang Q., Fang X., Wei X., Zhou Q., Zhang H., Wang M., Lu Z. (2022). Salt-induced hepatic inflammatory memory contributes to cardiovascular damage through epigenetic modulation of SIRT3. Circulation.

[39] Wang G., Yeung C.K., Wong W.Y., Zhang N., Wei Y.F., Zhang J.L., Yan Y., Wong C.Y., Tang J.J., Chuai M. (2016). Liver fibrosis can be induced by high salt intake through excess reactive oxygen species (ROS) production. J. Agric. Food Chem..

[40] Vaupel P., Multhoff G. (2021). Revisiting the Warburg effect: Historical dogma versus current understanding. J. Physiol..

[41] Wang Y., Liu X., Zhang C., Wang Z. (2018). High salt diet induces metabolic alterations in multiple biological processes of Dahl salt-sensitive rats. J. Nutr. Biochem..

[42] Tuttle K.R., Milton J.E., Packard D.P., Shuler L.A., Short R.A. (2012). Dietary amino acids and blood pressure: A cohort study of patients with cardiovascular disease. Am. J. Kidney Dis..

[43] Deng Y., Huang C., Su J., Pan C.W., Ke C. (2021). Identification of biomarkers for essential hypertension based on metabolomics. Nutr. Metab. Cardiovasc. Dis..

[44] da Silva A.A., do Carmo J.M., Li X., Wang Z., Mouton A.J., Hall J.E. (2020). Role of hyperinsulinemia and insulin resistance in hypertension: Metabolic syndrome revisited. Can. J. Cardiol..

[45] Mehrmohamadi M., Liu X., Shestov A.A., Locasale J.W. (2014). Characterization of the usage of the serine metabolic network in human cancer. Cell Rep..

[46] Tian Z., Liu Y., Usa K., Mladinov D., Fang Y., Ding X., Greene A.S., Cowley A.W., Liang M. (2009). Novel role of fumarate metabolism in dahl-salt sensitive hypertension. Hypertension.

[47] Kovarik J.J., Morisawa N., Wild J., Marton A., Takase-Minegishi K., Minegishi S., Daub S., Sands J.M., Klein J.D., Bailey J.L. (2021). Adaptive physiological water conservation explains hypertension and muscle catabolism in experimental chronic renal failure. Acta Physiol..

[48] Do M.H., Lee H.B., Lee E., Park H.Y. (2020). The effects of gelatinized wheat starch and high salt diet on gut microbiota and metabolic disorder. Nutrients.

[49] Gutierrez-Calabres E., Ortega-Hernandez A., Modrego J., Gomez-Gordo R., Caro-Vadillo A., Rodriguez-Bobada C., Gonzalez P., Gomez-Garre D. (2020). Gut microbiota profile identifies transition from compensated cardiac hypertrophy to heart failure in hypertensive rats. Hypertension.

[50] Kwan S.Y., Jiao J., Joon A., Wei P., Petty L.E., Below J.E., Daniel C.R., Wu X., Zhang J., Jenq R.R. (2022). Gut microbiome features associated with liver fibrosis in Hispanics, a population at high risk for fatty liver disease. Hepatology.

[51] Adams L.A., Wang Z., Liddle C., Melton P.E., Ariff A., Chandraratna H., Tan J., Ching H., Coulter S., de Boer B. (2020). Bile acids associate with specific gut microbiota, low-level alcohol consumption and liver fibrosis in patients with non-alcoholic fatty liver disease. Liver Int..

[52] Spencer M.D., Hamp T.J., Reid R.W., Fischer L.M., Zeisel S.H., Fodor A.A. (2011). Association between composition of the human gastrointestinal microbiome and development of fatty liver with choline deficiency. Gastroenterology.

[53] Jin K., Norris K., Vaziri N.D. (2013). Dysregulation of hepatic fatty acid metabolism in chronic kidney disease. Nephrol. Dial. Transplant..

[54] Li Y., Feng Y.F., Liu X.T., Li Y.C., Zhu H.M., Sun M.R., Li P., Liu B., Yang H. (2021). Songorine promotes cardiac mitochondrial biogenesis via Nrf2 induction during sepsis. Redox Biol..

[55] Poli A., Billi A.M., Mongiorgi S., Ratti S., McCubrey J.A., Suh P.G., Cocco L., Ramazzotti G. (2016). Nuclear phosphatidylinositol signaling: Focus on phosphatidylinositol phosphate kinases and phospholipases C. J. Cell. Physiol..

[56] Jin T., Song Z., Weng J., Fantus I.G. (2018). Curcumin and other dietary polyphenols: Potential mechanisms of metabolic actions and therapy for diabetes and obesity. Am. J. Physiol. Endocrinol. Metab..

[57] Au A., Cheng K.K., Wei L.K. (2017). Metabolomics, lipidomics and pharmacometabolomics of human hypertension. Adv. Exp. Med. Biol..

[58] Vallianou N., Christodoulatos G.S., Karampela I., Tsilingiris D., Magkos F., Stratigou T., Kounatidis D., Dalamaga M. (2021). Understanding the role of the gut microbiome and microbial metabolites in Non-Alcoholic Fatty Liver Disease: Current evidence and perspectives. Biomolecules.

