# Supplementary material for: Revealing the Pathogenesis of Salt-Sensitive Hypertension in Dahl Salt-Sensitive Rats through Integrated Multi-Omics Analysis
Source: Metabolites. 2022 Nov 7;12(11):1076. doi: 10.3390/metabo12111076 (PMC9694938; doi:10.3390/metabo12111076)
Supplement: Supplementary file 1 [file metabolites-12-01076-s001.zip › metabolites-1984027-supplementary.pdf]

Table S1. Microbial functions analysis by Phylogenetic Investigation of Communities by Reconstruction of Unobserved states (PICRUSt)

Function pathway	NS	HS	HSCUR	Raw p value	BH adjusted p-value
L3_Apoptosis	0.0216	0.058	0.0139	0.0151	0.0773
L3_Bacterial.chemotaxis	0.0583	0.0521	0.0429	0.0097	0.0773
L3_Bacterial.motility.proteins	0.0575	0.0523	0.0436	0.0257	0.0799
L3_Cell.cycle...Caulobacter	0.0509	0.056	0.0433	0.033	0.0814
L3_Flagellar.assembly	0.0618	0.0524	0.0414	0.0086	0.0773
L3_Peroxisome	0.0413	0.0591	0.0466	0.0234	0.0773
L3_Cell.division_Unclassified	0.0604	0.0536	0.0401	0.0171	0.0773
L3_Cell.motility.and.secretion_Unclassified	0.0514	0.0596	0.042	0.041	0.0879
L3_Inorganic.ion.transport.and.metabolism_Unclassified	0.0605	0.0531	0.0409	0.0172	0.0773
L3_Membrane.and.intracellular.structural.molecules_Unclassified	0.0605	0.0535	0.0406	0.0308	0.0814
L3_Other.transporters_Unclassified	0.0444	0.0563	0.0465	0.0213	0.0773
L3_ABC.transporters	0.0616	0.0505	0.0411	0.0173	0.0773
L3_Bacterial.toxins	0.1307	0.0163	0.0274	0.034	0.0814
L3_Cellular.antigens	0.0354	0.0558	0.0514	0.0301	0.0814
L3_G.protein.coupled.receptors	0.1979	0	0	0.0121	0.0773
L3_Ion.channels	0.0727	0.0519	0.0361	0.0044	0.0773
L3_MAPK.signaling.pathway...yeast	0.0242	0.0665	0.0516	0.0168	0.0773
L3_Phosphatidylinositol.signaling.system	0.0463	0.0582	0.0446	0.0291	0.0814
L3_Transporters	0.063	0.0489	0.0413	0.0218	0.0773
L3_Aminoacyl.tRNA.biosynthesis	0.0538	0.0546	0.0429	0.0358	0.0828
L3_Basal.transcription.factors	0.026	0.1435	0.0139	0.0232	0.0773
L3_Base.excision.repair	0.0566	0.0541	0.0409	0.0182	0.0773
L3_Chaperones.and.folding.catalysts	0.056	0.0545	0.0419	0.0211	0.0773
L3_Chromosome	0.0536	0.055	0.0428	0.0184	0.0773
L3_DNA.repair.and.recombination.proteins	0.0529	0.0555	0.0424	0.0196	0.0773
L3_DNA.replication	0.0503	0.0564	0.0431	0.0298	0.0814
L3_DNA.replication.proteins	0.0521	0.0557	0.0427	0.0234	0.0773
L3_Homologous.recombination	0.051	0.0561	0.043	0.0242	0.0785
L3_Mismatch.repair	0.0528	0.0553	0.0423	0.0229	0.0773
L3_Nucleotide.excision.repair	0.0529	0.0561	0.0418	0.0418	0.0888
L3_Proteasome	0.052	0.0554	0.043	0.0247	0.079
L3_Protein.export	0.0538	0.0552	0.0423	0.0191	0.0773
L3_RNA.degradation	0.0519	0.057	0.0426	0.0194	0.0773
L3_RNA.polymerase	0.0529	0.0554	0.0426	0.0351	0.0821
L3_RNA.transport	0.0536	0.0554	0.0426	0.0315	0.0814
L3_Replication..recombination.and.repair.proteins_Unclassified	0.0688	0.0503	0.0367	0.041	0.0879
L3_Ribosome	0.054	0.0551	0.0422	0.0194	0.0773
L3_Ribosome.Biogenesis	0.0543	0.0552	0.0423	0.0158	0.0773
L3_Ribosome.biogenesis.in.eukaryotes	0.0535	0.059	0.0397	0.0096	0.0773
L3_Sulfur.relay.system	0.0518	0.0575	0.0423	0.0395	0.0879
L3_Transcription.factors	0.0665	0.0481	0.0402	0.0227	0.0773
L3_Transcription.machinery	0.0536	0.0561	0.0422	0.0328	0.0814
L3_Transcription.related.proteins_Unclassified	0.1131	0.0347	0.0241	0.0058	0.0773

L3_Translation.factors	0.0527	0.0554	0.0428	0.0274	0.0814
L3_Translation.proteins_Unclassified	0.0552	0.0552	0.042	0.0156	0.0773
L3_Ubiquitin.system	0.0505	0.0604	0.0415	0.0404	0.0879
L3_Amino.acid.metabolism_Unclassified	0.091	0.0418	0.0306	0.0066	0.0773
L3_Amino.acid.related.enzymes	0.0565	0.0528	0.0425	0.0215	0.0773
L3_Aminobenzoate.degradation	0.0577	0.0559	0.0401	0.0118	0.0773
L3_Arginine.and.proline.metabolism	0.0596	0.0511	0.0421	0.0231	0.0773
L3_Ascorbate.and.aldarate.metabolism	0.0935	0.0389	0.0311	0.0328	0.0814
L3_Benzoate.degradation	0.0661	0.0469	0.0398	0.0436	0.0889
L3_Biosynthesis.of.vancomycin.group.antibiotics	0.0716	0.0491	0.0378	0.004	0.0773
L3_Biotin.metabolism	0.0517	0.0589	0.0414	0.0126	0.0773
L3_Carbon.fixation.pathways.in.prokaryotes	0.0526	0.0559	0.0428	0.037	0.0836
L3_Cysteine.and.methionine.metabolism	0.0546	0.0541	0.0424	0.0291	0.0814
L3_D.Glutamine.and.D.glutamate.metabolism	0.0586	0.0538	0.0409	0.003	0.0773
L3_Dioxin.degradation	0.088	0.0397	0.0343	0.0086	0.0773
L3_Drug.metabolism...other.enzymes	0.0569	0.0529	0.042	0.0048	0.0773
L3_Fatty.acid.biosynthesis	0.045	0.0564	0.0462	0.031	0.0814
L3_Fatty.acid.elongation.in.mitochondria	0.0001	0.1898	0.0098	0.0255	0.0799
L3_Flavone.and.flavonol.biosynthesis	0.1703	0.0054	0.0176	0.0322	0.0814
L3_Folate.biosynthesis	0.0552	0.0562	0.0414	0.0198	0.0773
L3_Glycan.biosynthesis.and.metabolism_Unclassified	0.0607	0.0546	0.0399	0.0211	0.0773
L3_Glycerolipid.metabolism	0.0644	0.0481	0.0412	0.0149	0.0773
L3_Glycerophospholipid.metabolism	0.0583	0.0536	0.0411	0.0139	0.0773
L3_Glycine..serine.and.threonine.metabolism	0.0545	0.054	0.043	0.0496	0.0973
L3_Glycosphingolipid.biosynthesis...ganglio.series	0.0071	0.067	0.0578	0.0464	0.0936
L3_Glycosyltransferases	0.0568	0.0545	0.0418	0.0397	0.0879
L3_Histidine.metabolism	0.0422	0.0569	0.0463	0.0217	0.0773
L3_Isoquinoline.alkaloid.biosynthesis	0.0642	0.0533	0.0387	0.0045	0.0773
L3_Lipoic.acid.metabolism	0.0515	0.0587	0.0421	0.033	0.0814
L3_Lipopolysaccharide.biosynthesis	0.0539	0.0587	0.0412	0.0296	0.0814
L3_Lipopolysaccharide.biosynthesis.proteins	0.0554	0.0568	0.0416	0.0436	0.0889
L3_Metabolism.of.cofactors.and.vitamins_Unclassified	0.0578	0.0564	0.0403	0.0114	0.0773
L3_Nitrotoluene.degradation	0.0756	0.0499	0.0334	0.002	0.0773
L3_Nucleotide.metabolism_Unclassified	0.0948	0.0409	0.0299	0.003	0.0773
L3_One.carbon.pool.by.folate	0.0554	0.0541	0.0421	0.0145	0.0773
L3_Pantothenate.and.CoA.biosynthesis	0.0544	0.0542	0.0428	0.0478	0.0956
L3_Penicillin.and.cephalosporin.biosynthesis	0.0529	0.0688	0.0349	0.0085	0.0773
L3_Peptidases	0.0568	0.0522	0.0427	0.0284	0.0814
L3_Photosynthesis	0.0526	0.0555	0.0427	0.0119	0.0773
L3_Photosynthesis.proteins	0.0528	0.0555	0.0427	0.0173	0.0773
L3_Porphyrin.and.chlorophyll.metabolism	0.0586	0.052	0.0416	0.0054	0.0773
L3_Protein.kinases	0.0605	0.0521	0.0409	0.0091	0.0773
L3_Purine.metabolism	0.0549	0.0542	0.0426	0.0231	0.0773
L3_Pyrimidine.metabolism	0.0525	0.0546	0.0432	0.0351	0.0821
L3_Pyruvate.metabolism	0.0557	0.0544	0.042	0.0183	0.0773
L3_Riboflavin.metabolism	0.0564	0.0554	0.0411	0.0362	0.0828
L3_Selenocompound.metabolism	0.0606	0.0521	0.0415	0.0043	0.0773
L3_Steroid.biosynthesis	0.0001	0.1895	0.01	0.0337	0.0814

L3_Stilbenoid..diarylheptanoid.and.gingerol.biosynthesis	0.0175	0.0578	0.0053	0.0097	0.0773
L3_Sulfur.metabolism	0.055	0.0562	0.0421	0.0134	0.0773
L3_Synthesis.and.degradation.of.ketone.bodies	0.0563	0.0594	0.0375	0.0062	0.0773
L3_Taurine.and.hypotaurine.metabolism	0.0585	0.0549	0.0408	0.0057	0.0773
L3_Terpenoid.backbone.biosynthesis	0.0506	0.057	0.0427	0.0284	0.0814
L3_Thiamine.metabolism	0.0543	0.0553	0.0422	0.0184	0.0773
L3_Various.types.of.N.glycan.biosynthesis	0.0336	0.116	0.0049	0.0432	0.0889
L3_Vitamin.B6.metabolism	0.0521	0.0555	0.043	0.0486	0.0962
L3_Xylene.degradation	0.0901	0.0406	0.0321	0.0031	0.0773
L3_Zeatin.biosynthesis	0.053	0.0551	0.0427	0.0207	0.0773
L3_beta.Lactam.resistance	0.0544	0.0654	0.0343	0.0117	0.0773
L3_Function.unknown_Unclassified	0.0586	0.0533	0.0414	0.0333	0.0814
L3_General.function.prediction.only_Unclassified	0.0567	0.0528	0.0423	0.0431	0.0889
